# Signatures of wave erosion in Titan’s coasts

**DOI:** 10.1126/sciadv.adn4192

**Published:** 2024-06-19

**Authors:** Rose V. Palermo, Andrew D. Ashton, Jason M. Soderblom, Samuel P. D. Birch, Alexander G. Hayes, J. Taylor Perron

**Affiliations:** ^1^ U.S. Geological Survey, St. Petersburg Coastal and Marine Science Center, St. Petersburg, FL, USA.; ^2^ MIT-WHOI Joint Program in Oceanography/Applied Ocean Science & Engineering, Cambridge and Woods Hole, MA, USA.; ^3^ Department of Geology and Geophysics, Woods Hole Oceanographic Institution, Woods Hole, MA, USA.; ^4^ Department of Earth, Atmospheric and Planetary Sciences, Massachusetts Institute of Technology, Cambridge, MA, USA.; ^5^ Department of Earth, Environmental, and Planetary Sciences, Brown University, Providence, RI, USA.; ^6^ Department of Astronomy, Cornell University, Ithaca, NY, USA.

## Abstract

The shorelines of Titan’s hydrocarbon seas trace flooded erosional landforms such as river valleys; however, it is unclear whether coastal erosion has subsequently altered these shorelines. Spacecraft observations and theoretical models suggest that wind may cause waves to form on Titan’s seas, potentially driving coastal erosion, but the observational evidence of waves is indirect, and the processes affecting shoreline evolution on Titan remain unknown. No widely accepted framework exists for using shoreline morphology to quantitatively discern coastal erosion mechanisms, even on Earth, where the dominant mechanisms are known. We combine landscape evolution models with measurements of shoreline shape on Earth to characterize how different coastal erosion mechanisms affect shoreline morphology. Applying this framework to Titan, we find that the shorelines of Titan’s seas are most consistent with flooded landscapes that subsequently have been eroded by waves, rather than a uniform erosional process or no coastal erosion, particularly if wave growth saturates at fetch lengths of tens of kilometers.

## Teaser

The coastline shapes of Titan’s seas are consistent with those of simulated and Earth coasts that have been eroded by waves.

## INTRODUCTION

**Fig. 1.**
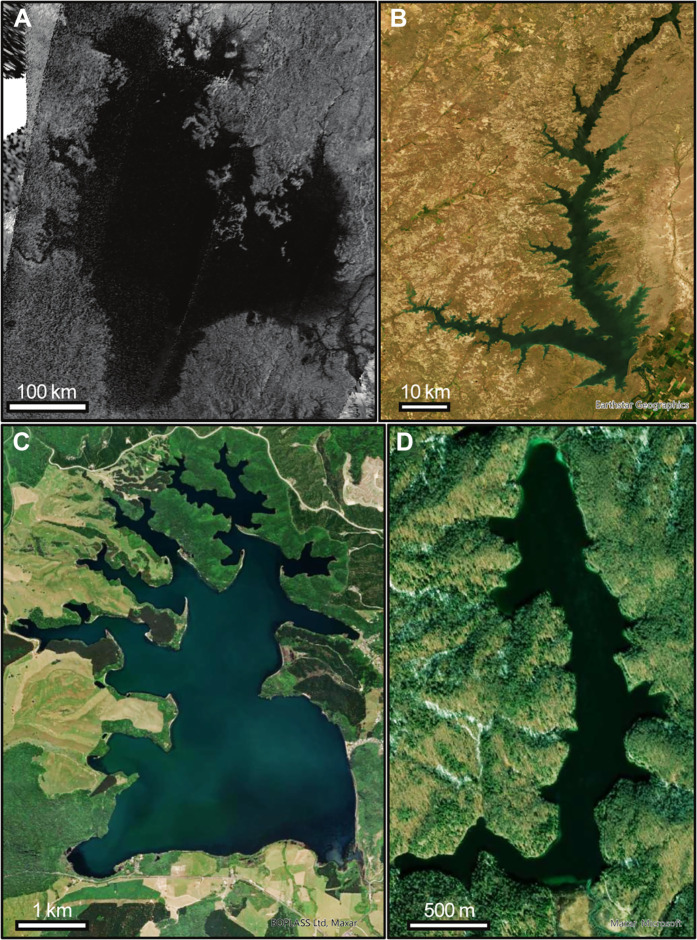
Lakes on Titan and Earth with coastlines shaped by a variety of erosional mechanisms. (**A**) Cassini SAR image of Ligeia Mare, Titan (NASA). (**B**) Fort Peck Lake, United States, a reservoir formed recently by flooding a landscape previously eroded by rivers [Map data: Esri World Imagery, Earthstar Geographics (*58*)]. (**C**) Lake Rotoehu, New Zealand, a lake in which flooded river valleys have been subsequently eroded by waves [Map data: Esri World Imagery, BOPLASS Ltd., Maxar (*58*)]. (**D**) Prošćankso Jezero, Croatia, a karst lake in which flooded river valleys have been eroded by dissolution [Map Data: Esri World Imagery, Maxar, Microsoft (*58*)].

Titan, Saturn’s largest moon, is the only known planetary body besides Earth on which standing liquids persist (Fig. 1A) (*1*). Hydrocarbon liquids, supplied by rainfall from Titan’s thick atmosphere, form rivers, lakes, and seas, most of which are found in the polar regions under Titan’s present climate (*2*). Titan’s lakes and seas have a variety of sizes and shapes, ranging from small, rounded lakes to large, complex seas that are comparable in size to the Great Lakes of North America (Fig. 1A) (*3*, *4*). Draining into Titan’s largest lakes and seas are actively flowing rivers (*5*, *6*) with lower reaches that appear to be flooded, forming embayments along the coasts (Fig. 1A) (*1*, *7*, *8*). These rivers are thought to have eroded Titan’s surface substantially, especially in the regions surrounding the seas (*9*–*11*).

Titan’s lakes and seas play prominent roles in the methane-based hydrologic cycle, and their shorelines may record the history of Titan’s climate interacting with its surface geology. To interpret that record, we must first understand what mechanisms have shaped the shorelines. Although several shoreline-shaping processes have been hypothesized to occur, the details of these processes and their relative influence on shoreline shape remain unknown. Crenulated, funnel-shaped embayments where rivers enter the northern seas (Fig. 1A) suggest that sea level rise has flooded river valleys that were carved into the landscape when sea level was lower (*1*, *2*, *12*), creating coastal features known as ria—similar in shape to embayments created by damming a river on Earth (Fig. 1B). In contrast, the absence of ria along the shoreline of the only large lake in Titan’s south polar region, Ontario Lacus, is consistent with the hypothesis that Milankovitch-like variations in Titan’s seasons cause surface liquids to cycle back and forth between the north and south polar regions, with the current orbital configuration producing a northern sea level highstand and a southern lowstand (*13*).

Additional mechanisms may have modified Titan’s shorelines after its river-eroded landscapes were flooded. For example, it has been suggested that Titan’s small north polar lakes were created and shaped by dissolution (*14*, *15*), whereas the large seas have been suggested to display evidence of mechanical erosion (*16*), possibly by waves (*3*). The combination of a thick, 1.5-bar atmosphere and large expanses of surface liquid could potentially lead to coastal erosion by wind-generated waves. Several lines of evidence support this idea. First, theoretical calculations and physical experiments indicate that waves could form on Titan’s seas (*17*, *18*). Second, Titan’s increased atmosphere-to-liquid density ratio, lower gravity, and lower surface tension of hydrocarbon liquids compared to water all suggest that the threshold winds required for wave generation should be lower than on Earth (*19*). Third, waves may have been observed on Titan’s seas in Cassini Visual and Infrared Mapping Spectrometer (VIMS) data as isolated patches of rough liquid surfaces (*20*). Fourth, specular reflections from Kraken Mare in VIMS observations could possibly be consistent with waves (*21*). Fifth, transient bright features in Ligeia Mare observed in synthetic aperture radar (SAR) images may also be evidence of surface waves (*22*, *23*). In addition, it has been suggested that waves have sculpted the long, smooth coast of Ontario Lacus, in Titan’s south polar region, not only because the smooth coastline resembles wave-dominated (yet depositional) Earth analogues but also because there appear to be shore-parallel features resembling relict shorelines, likely from earlier lake highstands and lowstands (*24*).

However, the evidence of waves on Titan remains indirect and scant. There are no in situ observations of liquid surfaces on Titan. Remote observations are sparse in space and time and do not cover all of Titan’s seasons, each of which spans ~7.5 Earth years (*25*), such that several studies specifically searching for signs of waves found none (*21*, *25*–*28*). Furthermore, a prior study has suggested that wave growth on Titan may saturate at fetch lengths—the distance over which the wind blows to generate waves—of several tens of kilometers due to a combination of slow wind speeds, the density of Titan’s atmosphere, and the density and viscosity of hydrocarbon liquids (*18*). Even with sufficient wind and distance to generate waves, settling aerosols could dampen wave formation on Titan’s liquid surfaces (*29*). It therefore remains unclear whether waves have modified the coasts of Titan’s seas.

If waves do form, then they could drive coastal erosion and sediment transport, both of which can alter coastline morphology (*30*, *31*). We focus our investigation on coastal erosion, analogous to the mechanisms that shape rocky coasts on Earth. As noted above, Titan’s most extensive coastlines—those of the northern seas—probably formed as rough, mountainous terrain were eroded by rivers and subsequently inundated by sea level rise (*4*). This suggests that the coastal environment is dominantly erosional, likely consisting of shore platforms and cliffs, with few or no depositional morphologies. We therefore ignore sediment deposition or reworking as a primary control on shoreline shape and assume that sediment produced by erosion is either too fine-grained to remain in the coastal zone or too volumetrically insignificant to influence coastline morphology. This does not exclude the possibility that sediment deposition and reworking may have influenced the morphology (*30*, *31*) of lowstand shorelines elsewhere on Titan, such as that of Ontario Lacus in the south polar region. The search for morphologic signatures of sediment redistribution along coastlines is left to future studies.

Previous efforts on Earth—and Titan—have yet to quantitatively identify coastline formation mechanisms from morphology alone. Earth lake shorelines formed by different mechanisms have statistically distinct fractal dimensions, but the differences are not sufficient to definitively identify lake shoreline formation mechanisms on Titan (*32*). Titan’s northern lakes can be statistically separated into four shape-based groups using elliptical Fourier descriptors, but these groups have not been associated with specific formation mechanisms (*33*).

Our approach is to focus on distinctive morphologic signatures produced by two possible mechanisms of coastal erosion: wave-driven erosion and uniform erosion. Wave-driven erosion in detachment-limited environments (in which erosion rate is limited by how rapidly cohesive material such as bedrock can be removed) occurs at a rate proportional to the wave power (*34*–*37*). In constrained basins, wave power is typically larger where waves can travel farther before reaching the coast (*38*). Therefore, wave-eroded coasts tend to exhibit smooth stretches of open coast, where more erosion occurs, and rougher sections in protected embayments, where less erosion occurs (Fig. 1C). Wave-driven erosion includes physical abrasion or removal of material by waves as well as any wave-enhanced erosion, such as dissolution enhanced by mixing due to wave activity—in the latter case, the erosion rate would still depend on the strength of the waves and how often they occur. The planform evolution of rocky coasts has been modeled as a function of coastal processes (*31*) and morphometrics (*39*, *40*).

A second mechanism that has been hypothesized to erode Titan’s coasts is uniform erosion, which encompasses a set of possible processes, including dissolution and backwasting (*14*, *41*). Titan’s crust consists mainly of water ice, but its surface solids may also include heavy hydrocarbon molecules, such as benzene, that are soluble in liquid methane and ethane, such that the liquid lakes and seas may slowly dissolve the solid coasts of the north polar terrain (*14*, *41*). Over long timescales, uniform erosion can be approximated as occurring at the same rate in all locations (*42*). Uniformly eroded coasts exhibit generally smooth shorelines, even within embayments, punctuated by sharp headlands (Fig. 1D).

Here, we test the hypothesis that coastal erosion has shaped Titan’s seas by investigating whether coastline shapes are most consistent with wave-driven erosion, uniform erosion, or no coastal erosion. Using a combination of landscape evolution modeling and wavelet-based measurements of coastline morphology, we identify and quantify morphologic signatures that can distinguish between three scenarios: (i) flooded, river-incised landscapes that have undergone no subsequent coastal erosion; (ii) coastal erosion by waves; and (iii) coastal erosion by a uniform process. Our results show that the morphologies of Titan’s northern seas are consistent with coasts that have been incised by rivers and subsequently eroded by waves. As on Earth, Titan’s coastlines can be leveraged to further unravel Titan’s climatic and geologic history.

## RESULTS

### Coastal erosion model

**Fig. 2.**
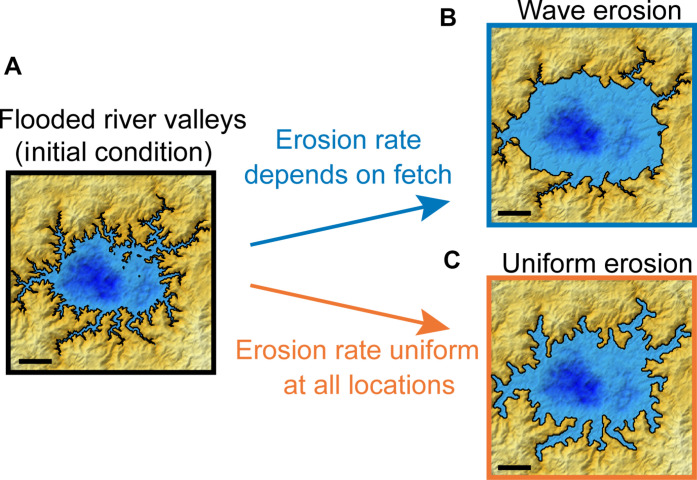
Distinct signatures of fluvial incision, wave erosion, and uniform erosion in numerical simulations of coastal erosion. Shaded relief maps with color indicating liquid depth (darker blues are deeper) and land surface height (lighter yellows are higher) and black lines tracing the shoreline. (**A**) Initial shoreline generated by raising sea level in a landscape previously incised by rivers. The shoreline evolves to different shapes if subjected to (**B**) wave-driven coastal erosion in which the erosion rate depends on fetch—the distance wind blows to generate waves—and the angle of approach of incident waves or (**C**) uniform coastal erosion in which the erosion rate is the same at all locations. Scale bars have the same nondimensional length (number of model grid cells and cell size) in all three panels.

To quantify the shoreline morphology produced by each erosional mechanism under idealized conditions, we simulated fluvial incision and coastal erosion of synthetic landscapes (Materials and Methods). Simulations were performed using the Numerical model of coastal Erosion by Waves and Transgressive Scarps (NEWTS) (*40*). To generate the initial coastlines, pseudo-fractal topographic surfaces were eroded to 94% of their initial relief with a terrestrial landscape evolution model (*43*), approximating the estimated erosion by rivers in Titan’s north polar region (*9*, *11*), then flooded to simulate sea level rise, creating lakes with dendritic ria embayments (Fig. 2A). The shorelines of these lakes were then eroded by either wave-driven or uniform coastal erosion until lake areas increased 50% relative to their initial size (Fig. 2, B and C). Under wave-driven erosion, the coast retreats at a rate that depends on the local fetch assuming an isotropic wind field. Under uniform erosion, the coast retreats at the same rate everywhere. Sea level is constant in our simulations. When comparing simulation results with shorelines on Earth or Titan, this assumes either constant sea level or erosion during repeated episodes of similar sea level.

The two different erosional mechanisms generate visually obvious differences in shoreline morphology (Fig. 2 and fig. S1). Operating on the initial coastlines with numerous ria, wave erosion smooths exposed stretches of coast, which have large fetch, while the roughness in embayments and other protected stretches of coast is preserved due to the small fetch, a phenomenon particularly evident in crenulations at the tips of ria (Fig. 2B). In contrast, uniform erosion widens embayments and smooths small-scale roughness along most sections of coast regardless of fetch, except for headlands, which become sharpened into crenulated, thick-necked points that jut into the main basin (Fig. 2C) (*42*). These distinct shoreline morphologies—particularly the different relationships between shoreline roughness and fetch—serve as potential fingerprints of wave erosion and uniform erosion in natural coastlines.

### Fingerprinting coastal processes

**Fig. 3.**
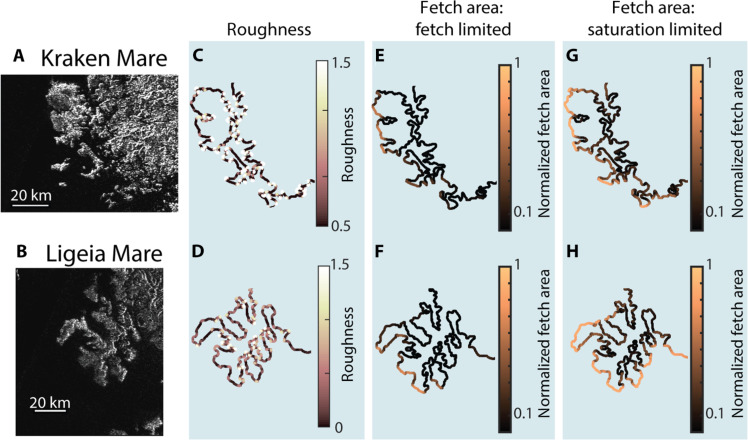
Comparison of roughness and fetch area for two example sections of Titan’s sea coasts. NASA Cassini SAR images of stretches of coast along (**A**) Kraken Mare and (**B**) Ligeia Mare. Subsequent columns show corresponding maps of (**C** and **D**) shoreline roughness, (**E** and **F**) normalized fetch area assuming waves are fetch-limited, and (**G** and **H**) normalized fetch area assuming a saturation fetch length of 20 km. See Fig. 4 for regional maps of Kraken Mare and Ligeia Mare.

Quantifying these differences in shoreline morphology requires a fingerprinting technique based on local information because both wave erosion and uniform erosion result in localized regions of high roughness; a global approach such as calculating fractal dimension (*32*) would encounter difficulties distinguishing spatial differences in roughness. Accordingly, we developed a technique focusing on local relationships between shoreline roughness and fetch area (Materials and Methods). First, we unwrap the closed shoreline shape into a Cartesian function by calculating the azimuth from each shoreline point to the next as a function of distance along the shoreline (fig. S2, A to C), detrending this function, and then integrating the detrended azimuth with respect to distance (fig. S2E). We then compute the wavelet power spectrum of the unwrapped shoreline, which gives a measure of local coastline shape as a function of scale (wavelength) and location (fig. S3A). Using scales coarser than the grid scale but finer than the overall lake shape (which depends more on the background topography than on coastal processes), we compute the deviation of the local wavelet power spectrum from the global (spatially averaged) wavelet power spectrum (Materials and Methods; fig. S3). This quantity measures how much more or less shoreline position varies at each location on the shoreline relative to the rest of the lake over scales relevant for coastal processes. We refer to this morphologic quantity as the “roughness” (Fig. 3). Simply put, a lower roughness means a smoother stretch of shoreline compared to the rest of the lake, and a higher roughness means a comparatively rough stretch of shoreline.

To identify the fingerprint of each erosion process on coastal morphology, we compare the shoreline roughness with a morphologic proxy for the time-integrated wave power incident to each stretch of coastline. For a given wind event, wave power depends on wind speed and is proportional to the fetch, the distance over which the winds generate waves. Fetch is controlled either by the size of the basin or, if the basin is large, the distance over which the waves become saturated, or the maximum storm size (*38*). If waves continue to grow as long as fetch length increases, then the system is fetch-limited, and no maximum is imposed on fetch length. If waves only grow up to a certain fetch length and then saturate, then the system is saturation-limited, and the fetch length in all directions is truncated to a maximum value. When waves reach the coast, wave power per unit shoreline length depends on the angle between the wave propagation direction and the shoreline orientation (Materials and Methods) (*38*, *40*).

Without measurements of wind speed and direction on Titan, we focus on how coastline shape may create differences in wave power along the coast by assuming a wave climate characterized by isotropic winds of uniform strength. Over time, each point along the coastline eventually is exposed to waves generated over a given area of open sea that can be “seen” from that point (fig. S4) (*40*). This time-integrated shore-normal wave power can be approximated by a weighted fetch area that includes the effects of shoreline orientation and can be adjusted for wave saturation by imposing an upper limit to fetch length (fig. S4; Materials and Methods). Last, to facilitate comparisons between lakes of different size, we normalize the weighted fetch area by the maximum value for the entire lake (Fig. 3). The normalized fetch area thus serves as a proxy for the time-averaged wave power a single shoreline point experiences relative to the highest value for the lake.

River incision, wave erosion, and uniform erosion should each generate distinct relationships between shoreline roughness and normalized fetch area. The river-incised coastlines that serve as the initial condition in our simulations (Fig. 2A) may begin with a weakly negative relationship because ria (which have small fetch areas) may be rougher than the intervening sections of coast generated by flooding a pseudo-fractal surface (which have larger fetch areas). Wave-eroded shorelines should have a stronger negative relationship between roughness and normalized fetch area because more erosion and smoothing occur on exposed sections of coast where normalized fetch area is larger (Fig. 2B). Uniform erosion should generate a weakly positive relationship between roughness and fetch area because it smooths the coastline indiscriminately; however, this relationship is not as straightforward due to the higher roughness on sharpened headlands, where normalized fetch area is greatest (Fig. 2C). We also compare shorelines from these three detachment-limited erosional scenarios with elevation contours from a relatively smooth red noise surface (Materials and Methods). The goal of this analysis is to test if our landscape evolution model results can be distinguished from a smooth, random terrain that was flooded.

Analyses of the numerical simulations of coastal erosion support the hypothesis that shoreline roughness and normalized fetch area can be used to fingerprint wave-driven and uniform erosion and distinguish them from a coastline consisting only of flooded river valleys, consistent with the visual differences in Fig. 2. The ratio of shoreline roughness to normalized fetch area takes on statistically distinct distributions for the three cases of initial conditions with ria, wave erosion, and uniform erosion in 2305 simulated shorelines and for 405 contours from the smoother initial surface [*P* << 10^−6^ in a Kruskal-Wallis (KW) one-way analysis of variance test; table S2].

We further quantify the different signatures of the three coastal erosion scenarios using the joint probability distribution function (JPDF) of roughness and fetch area (figs. S5 and S6; Materials and Methods). We use the JPDF, rather than simply examining the correlation between roughness and fetch area, because coastal erosion additionally alters the fraction of the shoreline that takes on certain values of roughness and fetch area—smoother shorelines are shorter than rough shorelines. We construct a characteristic JPDF for each of the three cases by compiling a two-dimensional (2D) histogram of roughness versus normalized fetch area for all shoreline points across all 2305 simulations (fig. S5).

**Fig. 4.**
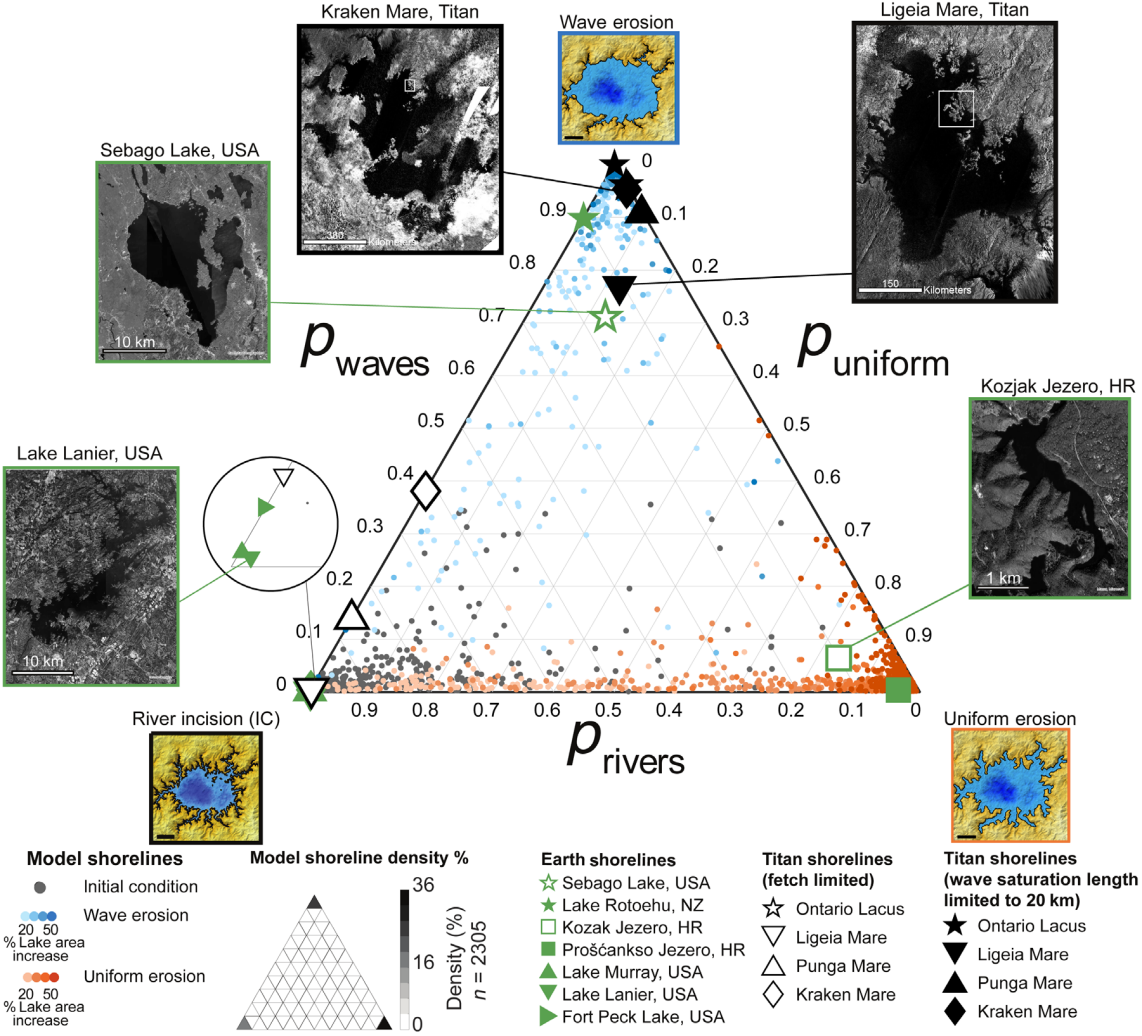
Ternary classification diagram of dominant coastal erosion mechanism. Axes are the categorical probabilities that a shoreline was eroded dominantly by rivers alone (*p*_rivers_), fetch-dependent wave erosion (*p*_waves_), or uniform erosion (*p*_uniform_), as determined by a comparison of the shoreline’s JPDF of roughness and fetch area with those of model simulations. Images of example simulations from Fig. 2 are placed at the corners to aid interpretation of the diagram. Individual model simulations of wave erosion (blue circles) and uniform erosion (orange circles) are plotted, with point opacity indicating cumulative lake area increase due to erosion. Gray circles represent simulation initial conditions (IC) with shorelines eroded only by rivers. The density of the 2305 model shoreline points is shown as an inset in the legend, demonstrating that most model simulations are correctly categorized by driving mechanism with >90% categorical probability. Titan shorelines are plotted for two scenarios: fetch-limited waves (waves can grow across the entire sea; black open symbols) and saturation-limited waves (waves cease to grow at a fetch length of 20 km; black closed symbols). The fetch-limited Ontario Lacus data point is partially covered by the saturation-limited Kraken Mare data point. White boxes in NASA Cassini SAR images of Ligeia Mare and Kraken Mare indicate the locations shown in Fig. 3. Shorelines on Earth are plotted as green symbols, with inset aerial images of three examples [Map data: Esri World Imagery, Earthstar Geographics, Maxar, Microsoft (*58*)] and symbol shape indicating known dominant erosional mechanisms (stars, wave erosion; squares, dissolution; and triangles, river incision). Earth examples assume fetch-limited conditions due to small lake size. Earth examples are found in the United States (USA), Croatia (HR), and New Zealand (NZ). Ternary plot generated using Ternplot (*59*).

Given an observed shoreline for which the dominant erosional process is unknown, we estimate the likelihood that the observed shoreline was eroded by each of the three end-member mechanisms (river incision alone, wave erosion, or uniform erosion) by comparing the JPDF of the observed shoreline with the characteristic JPDFs of the simulated shorelines (Materials and Methods). This yields a set of three categorical probabilities, which can be plotted on a ternary diagram with the three end-member mechanisms at the corners (Fig. 4). Testing this approach with the individual simulation results, we find that categorization accuracy increases as shorelines progressively erode, with most shorelines being correctly classified with >95% probability once lake area has increased to 150% of its initial value (Fig. 4).

To test the sensitivity of this result to the assumption of an isotropic wind climate, we ran 48 simulations in which coastal erosion under an anisotropic wave climate caused a 50% lake area increase (Materials and Methods). The ratio of shoreline roughness to normalized fetch area for shorelines created by waves in an isotropic wind climate is not statistically distinct from the ratio for an anisotropic wind climate (*P* = 0.12 in a KW one-way analysis of variance test; table S3). Furthermore, all 48 model simulations conducted with an anisotropic wave climate were correctly categorized as wave-eroded, indicating that the results of our analysis are not strongly sensitive to wind direction for the amount of cumulative coastal erosion investigated here.

### Application to Earth and Titan

We further tested our classification method on Earth, where the processes shaping shorelines can be determined independently. We mapped seven Earth lakes (fig. S7; Materials and Methods) and calculated their JPDFs (fig. S8), assuming fetch-limited conditions due to the relatively small size of the examples used. Analysis of three reservoirs, Lake Murray, Lake Lanier, and Fort Peck Lake, indicates >90% probability that each shoreline is consistent with a landscape that was incised by rivers and then flooded, as expected for artificial reservoirs (Fig. 4). The shorelines of two lakes where waves are an important morphological agent, Sebago Lake and Lake Rotoehu, are consistent with model shorelines that have undergone wave erosion with >90% probability (Fig. 4). Last, two lakes found in a karst landscape where dissolution is an important mechanism, Prošćansko Jezero and Kozjak Jezero, demonstrate a >70% probability that their shorelines are consistent with model simulations of uniform erosion (Fig. 4). Thus, mapped shorelines on Earth demonstrate that our classification method can identify the known erosional processes that have shaped the shorelines, giving confidence that the method can be applied to Titan, where ground truthing is not possible.

On Titan, there are no in situ measurements of the wave climate, so we analyze planform maps of coastlines to determine if Titan’s coasts contain morphologic evidence of wave erosion. We mapped the coastlines of three seas and one large lake from Cassini SAR images (Fig. 3 and fig. S9; Materials and Methods). We focused on Titan’s north polar seas, Ligeia Mare, Punga Mare, and Kraken Mare, which are likely to have dominantly erosional coastlines for the reasons noted in Introduction. We excluded the small north polar lakes as their formation mechanism remains unclear. We included Ontario Lacus, the largest lake in Titan’s southern polar region, for comparison with the results for the north polar seas, noting that possible depositional features along its shoreline (*24*) may be a sign of coastal mechanisms that deviate from those assumed in our erosional model.

As with the lakes on Earth, we used the maps of Titan coastlines to calculate shoreline roughness and normalized fetch area, from which we constructed JPDFs for the Titan shorelines (fig. S10). Given the large size of Titan’s seas and the lack of direct observations of wave growth, we consider both fetch-limited and saturation-limited scenarios. In the fetch-limited scenario, our analysis indicates >90% probability that the south polar lake, Ontario Lacus, is consistent with wave-eroded models, whereas the north polar seas yield <30% probability of consistency with wave-eroded models and >70% probability of consistency with model initial conditions (flooding of a landscape that has been eroded by rivers alone, with no subsequent coastal erosion) (Fig. 4). Assuming saturation-limited conditions with maximum fetch lengths of 20 km, we find that the shorelines of Kraken Mare, Punga Mare, Ligeia Mare, and Ontario Lacus are all consistent with wave-eroded model shorelines with >70% probability (Fig. 4). Results are similar for saturation lengths up to several tens of kilometers (fig. S11). Thus, we find stronger support for wave erosion on Titan if wave growth is saturation-limited, a scenario that seems likely given the factors noted above. The statistical support for wave erosion of coastlines in Titan’s largest liquid reservoirs is consistent with the qualitative observation that more exposed stretches of coast are smoother than protected sections along many stretches of Titan’s sea coasts (Fig. 3).

Our results suggest that the coastlines of Titan’s largest liquid bodies are most consistent with shorelines that have been modified by wave erosion and river incision. The four Titan coasts we analyzed show <5% probability of uniform erosion when considering the saturation-limited scenario and <20% probability when considering the fetch-limited scenario. Therefore, our results suggest that the largest seas and lakes are not consistent with erosion by uniform processes (i.e., dissolution), as previously hypothesized for some of Titan’s landscapes (*3*, *14*–*15*, *41*).

## DISCUSSION

Our numerical model results show that river incision, wave erosion, and uniform erosion produce distinct shoreline morphologies (Fig. 2 and fig. S1) that can be quantitatively distinguished using the joint probability distribution of shoreline roughness and normalized fetch area (fig. S5). This fingerprinting method correctly identifies the dominant coastal erosion mechanism in 96% of simulated shorelines and all seven of the mapped Earth shorelines (Fig. 4). On Titan, we find that the coastlines of the northern seas and Ontario Lacus are more consistent with erosion by wind-generated waves than with uniform erosion by dissolution or scarp retreat (Fig. 4).

Our results do not prove that waves form on Titan—this would require direct observations—but they do show that, if erosion has altered Titan’s sea coasts, the shoreline shapes are more consistent with wave-driven erosion than with uniform erosion due to dissolution or scarp retreat. This consistency is generally true for both fetch-limited and saturation-limited scenarios (Fig. 4 and fig. S11). The relationship between shoreline roughness and fetch for Ontario Lacus, in Titan’s south polar region, is consistent with wave-eroded coastlines regardless of the fetch limit (Fig. 4 and fig. S11). Titan’s north polar seas fall along the continuum between flooded, river-incised shorelines and wave-eroded shorelines for most fetch scenarios but align more closely with flooded, river-incised landscapes if fetch-limited conditions are assumed (Fig. 4). This correlation occurs because the largest differences in roughness under saturation-limited conditions are expected between small embayments and any open part of the coast, as the open parts of the coast all incur a similar amount of wave power (Fig. 3). In contrast, under fetch-limited conditions, differences in roughness among exposed sections of coast are expected because the most open portions of the coast encounter more wave energy than moderately exposed sections. However, even under the less likely assumption that waves grow over the entire length of the seas (up to hundreds of kilometers), there remain 15 and 40% probabilities that the coastline shapes of Punga Mare and Kraken Mare, respectively, are consistent with wave erosion. It is also important to note that the fingerprints of coastal erosion processes become stronger, and our morphologic technique for detecting those fingerprints becomes more reliable, as the coast erodes more (provided the coast does not erode so much that it becomes nearly circular), as shown by analysis of progressively eroding model simulations (Fig. 4). The lower probabilities of identifying wave erosion for fetch-limited scenarios could mean that Titan’s sea coasts have not eroded as much as the coasts in our simulations (50% increase in lake area), possibly indicating that recent sea level rise has outpaced the morphological signature of wave erosion.

The saturation fetch length on Titan, if wind-generated waves do form there, is unknown. Theoretical models of wind climate (*44*) and waves (*19*) suggest that waves on Titan may saturate at fetch lengths as short as 1 km or up to tens of kilometers (*18*). With a conservatively large saturation fetch length of 20 km, all the Titan seas we mapped are most consistent with wave erosion (Fig. 4). Shorter saturation fetch lengths generally result in even closer alignment with wave erosion (fig. S11). The only scenario in which shoreline morphology is partly consistent with uniform erosion is for Ligeia Mare if the saturation fetch length is assumed to be ~10 km or less (fig. S11). If wave erosion has shaped Titan’s sea coasts, then our analysis suggests that the saturation fetch length is a few tens of kilometers or less, consistent with previous predictions (*18*), or that the erosion has increased the area of the seas by less than 50%.

Surface wind directions and magnitudes are poorly constrained on Titan. The model framework presented here could be used to explore the role of various wind directions and wave magnitudes on the coastal morphology of Titan’s north polar seas, potentially enabling landform-based estimates of Titan’s surface winds. However, our results show that, when wave erosion has increased lake area by 50%, an anisotropic distribution of wind directions does not prevent our statistical method from correctly identifying the morphologic signature of wave erosion (table S3). The conditions under which shoreline shape can be used to probe the distributions of wind direction and strength is a topic for future investigation.

Our approach assumes that shoreline shape only changes because of erosion; it ignores coastal features created by transport and deposition of sediment. This simplifying assumption does not interfere with our ability to correctly identify the processes influencing erosional lake coasts on Earth (Figs. 1, B to D, and 4); some of which contain clearly depositional shoreline reaches. The assumption of detachment-limited sea coasts around Titan’s northern seas, while not testable at present, is reasonable for three reasons. First, depositional landforms appear to be rare—only one major coastal depositional feature has tentatively been identified on Titan, a possible river delta on Ontario Lacus at Titan’s south (*24*). Second, Titan’s sediment is probably more buoyant in hydrocarbon liquids than Earth’s sediment is in water (*6*, *8*, *45*–*47*), so sediment might be removed from the coastal system more easily. Third, Titan’s north polar seas are thought to be in highstand conditions (*13*), flooding steep topography. Altimetry data show depths exceeding 100 m in Ligeia Mare (*48*) and Kraken Mare (*49*), and SAR images show what appear to be flooded canyons in nearshore areas (*5*). Low-relief coastal plains, which facilitate creation and preservation of depositional sedimentary features, may be more deeply submerged at present. The complex morphology in the submerged terrain, including deep, flooded canyons (*5*), suggests that differences in localized wave shoaling and refraction along the coast could be important. Future work could explore the role of nonlinear wave transformations on Titan’s coastal environment.

Such coastal plains may currently be exposed in the south polar region, where sea level is thought to be near a lowstand (*13*). This could be why our technique consistently classifies Ontario Lacus as purely wave-eroded: Its open stretches of coast may be smooth because they are lowstand features composed of reworked seabed sediment. This scenario is consistent with the presence of possible depositional features along the coast of Ontario Lacus but not the northern seas (*1*). The coastline of Ontario Lacus may bear the morphologic signature of waves, but their smoothness could be a signature of wave-driven sediment transport rather than, or in addition to, wave erosion.

Our analysis of large lakes and seas allows for the possibility that Titan’s smaller lakes are shaped by different coastal processes. For example, it has been proposed that many of the small lakes in the north polar region formed through karst-like dissolution of a surface-mantling material that is soluble in hydrocarbons (*3*, *14*, *15*). Our study does not test that hypothesis, although our approach could, in principle, be used to investigate erosional processes in the smaller lakes. We chose to focus on Titan’s largest lakes and seas because they are thought to have formed by inundation of preexisting topographic depressions (*4*, *15*, *25*), allowing fingerprints of coastline erosion to be more easily identified.

The evidence of wave erosion in the morphology of Titan’s northern seas is somewhat unexpected given theoretical predictions of slow wind speeds (*50*) and small wave heights (*18*, *19*, *50*). Our results give additional confidence to the evidence for waves from remote observations (*20*–*23*, *27*). With estimated typical wind speeds of ~1 m/s (*50*, *51*), simple wave models suggest that fair weather waves on Titan would be only a few tens of centimeters in height, with a peak closer to 1 m in stronger summer winds (*18*, *19*). Although waves of this size could cause coastal erosion, the morphological signature of wave erosion identified here suggests that climate scenarios with occasional stronger winds, occurring in some models (*52*) both regionally and locally, are also plausible. Formation of wave-cut coastal platforms could have preserved evidence of past liquid levels on Titan (*3*, *15*), and these markers of past equipotential surfaces could also record subsequent crustal deformation (*53*, *54*). The various landforms produced by coastal erosion would be high-priority targets for future Titan orbiter and lander missions.

## MATERIALS AND METHODS

### Coastline evolution model

Synthetic initial coastlines were generated by allowing rivers to erode a low-relief, randomly rough topographic surface and then partially flooding the resulting landscape, mimicking the sequence of processes that is thought to have occurred on Titan. Random topographic surfaces were generated by computing doubly periodic red noise with vertical relief comparable to Titan’s north polar terrain (*55*) and superimposing an inverted cosine function to create a closed depression near the center of the model domain (*40*). For landscape evolution model red noise, we used a power spectrum slope of −1.6, and for the smoother initial surface, we used power spectrum slopes of −1.8 and −2.0. For both, we used an elevation variance of 10,000 m^2^. We then used a landscape evolution model (*43*) to simulate bedrock river incision according to the stream power law. Model parameters for the river incision simulations are listed in table S1. River incision was terminated after it had reduced the relief, which we define as the difference between the mean and minimum elevation in the landscape, by 6% (*9*).

Coastal erosion of synthetic river-incised landscapes was modeled using NEWTS1.0 (*40*), a cellular model of detachment-limited shoreline erosion. Coastal erosion occurred either at a rate depending on shore-normal wave energy flux (a function of fetch and shoreline orientation), representing wave-driven erosion, or at a constant rate along the coast, representing a uniform erosional process such as dissolution or backwasting. Input parameters for the coastal erosion simulations are listed in table S1. Model runs were terminated after coastal erosion increased the lake area by a specified percentage, typically 50%. This amount of coastal erosion was chosen to be sufficient to impart a morphologic signature of the dominant erosional process, but not so much that coastlines took on a nearly circular shape with little morphologic variability.

### Shoreline mapping

Shorelines were extracted from the simulated landscapes by tracing the four-connected boundary of land cells in contact with liquid cells (fig. S4) using the software in NEWTS (*40*). Shorelines on Titan were mapped by hand at 1:150,000 resolution. Titan shorelines were interpreted to be the boundary between relatively SAR-bright (high backscatter) and SAR-dark (low backscatter) pixels in Cassini SAR images, a boundary that can be several pixels across and therefore difficult to discern (fig. S9). To mitigate this uncertainty, Ligeia Mare was mapped three times, and the local shoreline most consistent among the three mapped shorelines was chosen for analysis. The other shorelines were mapped once following criteria consistent with the consensus Ligeia Mare map. Our shoreline mapping is broadly consistent with previous works [e.g., (*4*)]. Earth shorelines were extracted from either Sentinel-2 imagery (10-m resolution) or the Google Earth Surface Water dataset [30-m resolution; (*56*)] using threshold values to identify liquid and land (fig. S7). A list of data sources and threshold values used for Earth lakes is provided in table S4.

### Quantifying shoreline morphology

We quantified shoreline morphology using wavelet transforms, which measure variability in shoreline shape as a function of spatial scale (wavelength) and location. First, each shoreline was “unwrapped” by measuring the azimuth between vectors connecting consecutive shoreline points as a function of alongshore distance (fig. S2, A to C) and then integrating this azimuth function with respect to distance. This yields a periodic Cartesian function representing shoreline shape as a single-valued function of alongshore distance (fig. S2D), which we refer to as the “unwrapped shoreline.” We computed the wavelet power spectrum of the unwrapped shoreline (fig. S3A) using the Morlet wavelet, which yields wavelet scales that are approximately equal to sinusoidal Fourier wavelengths (*57*). We then normalized the wavelet power spectrum to the global wavelet power spectrum (fig. S3, B and C) to remove the trend of increasing spectral power with increasing spatial scale, which makes it more straightforward to compare shoreline shape across different spatial scales (short and long wavelengths)

$${\tilde{P}}_{\mathrm{ij}}=\frac{{|W_{\mathrm{ij}}|}^{2}}{\frac{1}{N_{x}}\sum_{i}{|W_{\mathrm{ij}}|}^{2}}$$ (1)

where ∣*W_ij_*∣ is the wavelet amplitude at the *i*th shoreline location for the *j*th wavelet scale (hereafter referred to as wavelength), *N_x_* is the number of shoreline points, and $\tilde{P}$ is the normalized wavelet power. The sum of the normalized wavelet power across a specified range of wavelengths, which is analogous to the variance of the input function within that wavelength range if computed from the un-normalized wavelet spectrum, provides a measure that we term the local shoreline roughness at point *i* (σ*_i_*). We calculated the shoreline roughness for each shoreline point as (*57*)

$$\sigma_{i}=\frac{\delta j\delta x}{C_{\delta }N_{x}}\sum_{j}\frac{{\tilde{P}}_{\mathrm{ij}}}{\lambda_{j}}$$ (2)

where δ*x* is the alongshore spacing between shoreline points, λ*_j_* = *s*_0_2^*j*δ*j*^ is the *j*th wavelength, with δ*j* = 0.25 in our calculations, *C*_δ_ = 0.776 is the reconstruction factor for the Morlet wavelet, and *s*_0_ = 2δ*x* is the smallest wavelength computed in the wavelet power spectrum.

We focused on wavelengths that are longer than the grid scale but shorter than major coastline features that are likely dictated by the landscape’s geologic structure. These intermediate wavelengths represent the scales over which coastal erosional processes have their strongest influence on shoreline morphology and thus are most likely to produce observable signatures. To standardize comparisons among shorelines with different shapes and sizes, we calculated the roughness using a minimum wavelength that corresponds to the 5th percentile of the roughness and a maximum wavelength that corresponds to the 50th percentile of the roughness. The reported roughness is a measure of the variability in the unwrapped shoreline position over this range of wavelengths for each alongshore point.

### Fingerprinting coastal erosion processes

We developed a statistical procedure that can reliably identify the dominant coastal erosion mechanism in simulated and observed erosion-dominated shorelines based on measurements of shoreline morphology. The procedure uses the relationship between fetch and shoreline roughness, which differs between the three end-member erosional scenarios considered here: flooded, river-incised landscapes that have undergone no subsequent coastal erosion (Fig. 2A); coastal erosion by waves (Fig. 2B); and coastal erosion by a uniform process (Fig. 2C). The angle-weighted fetch area for each shoreline point—a proxy for wave power per unit distance along the coast—was computed using the software in NEWTS as the angular integral of the fetch length extending in each direction from each shoreline location and weighted by the cosine of the angle between the fetch direction and the direction normal to the coast (fig. S4) (*40*). In cases that used a maximum saturation fetch length, a maximum fetch length was imposed. The normalized fetch area for each shoreline point is the angle-weighted fetch area for that point divided by the maximum for the lake.

For each shoreline, we estimated the JPDF of roughness and normalized fetch area by constructing a 2D histogram for all the points along the shoreline (fig. S6). The characteristic JPDF for coasts shaped by river incision with no subsequent coastal erosion was obtained by constructing a cumulative histogram for all initial coasts used in the model simulations (fig. S5D), and the JPDFs for the two erosional processes (wave-driven and uniform erosion; fig. S5, E and F) were obtained by constructing cumulative histograms for all model runs that used each erosional process, with shorelines measured after a 20, 30, 40, and 50% increase in lake area.

As an initial test of whether the distributions of fetch area and roughness for the three process scenarios are distinct, we performed a KW test on model morphological metrics from each process. KW tests for (i) normalized fetch area, (ii) roughness, and (iii) normalized fetch area divided by roughness each resulted in a rejection of the null hypothesis that the morphologic data resulting from each process come from the same distribution (table S2). The summary statistics for the KW test on roughness, normalized fetch area, and roughness divided by normalized fetch area illustrate the distinctions between the distributions for the three coastal erosion process scenarios (table S2). We then built a framework for measuring the morphologic similarity between a mapped shoreline and the three end-member process scenarios based on a comparison of the JPDF for the mapped shoreline with the characteristic JPDFs for the end-member erosional process scenarios (fig. S4). We computed the difference between any single mapped shoreline’s JPDF (*p*_shoreline_) and the modeled characteristic JPDF for process *i* (*p*_process,*i*_) using the Kullback-Leibler (KL) divergence

$${\text{KL}}_{i}=\text{KL}\left(p_{\text{shoreline}}\|p_{\text{process},i}\right)=\sum p_{\text{shoreline}}\text{log}\frac{p_{\text{shoreline}}}{p_{\text{process},i}}$$ (3)

where KL*_i_* is the KL divergence of a given individual shoreline for the *i*th process. The minimum KL divergence represents the best-fitting erosional process scenario. To estimate the probability that a mapped shoreline matches each end-member process scenario, we modeled the three probabilities (one for each process) as a categorical distribution over the three processes and compute the probabilities as the softmin over the KL divergences, scaled by a learned parameter α

$$P\left(\text{process}=i|p_{\text{shoreline}}\right)=\frac{e^{-\alpha {\text{KL}}_{i}}}{\sum_{i}e^{-\alpha {\text{KL}}_{i}}}$$ (4)

We chose the parameter α to minimize the categorical cross- entropy between the true labels of the model data (the actual erosion process that shaped the model shoreline) and the categorical probabilities for each model shoreline. The value of α was fit using a dataset of models eroded to a 20, 30, 40, and 50% increase in lake size to capture variability in shape due to different amounts of erosion. The cross-entropy is defined as

$$\text{cross-entropy}=-\sum_{j}\text{log}P\left(y_{j}|p_{\text{shoreline},j}\right)$$ (5)

where *p*_shoreline,*j*_ is the JPDF for the *j*th shoreline, and *y_j_* is the process that shaped the *j*th shoreline. Minimizing the cross-entropy in this way finds the categorical distribution that best matches the distribution of the model data.

Applying this framework to all the simulated shorelines shows that our approach predicts (assigns the highest probability to) the correct erosional scenario with 96% accuracy (Fig. 4). The same α value was then used to assign categorical probabilities to mapped shorelines based on the JPDFs for shorelines on Earth (fig. S8) and Titan (fig. S10), yielding the values plotted in Fig. 4 and fig. S11.

Most coastal erosion simulations assumed an isotropic distribution of wind direction and strength. To test the sensitivity of our results to the assumed distribution of winds, we performed additional simulations with an asymmetric wind climate, in which the fetch was weighted by the function *B*cosθ, where *B* = 0.5 and θ is the wind direction. The summary statistics for the KW tests on all three roughness metrics demonstrate that it is not possible to reject the null hypothesis that the measurements from simulated shorelines with isotropic and anisotropic winds come from the same distribution (table S3). Furthermore, the 48 model simulations conducted under anisotropic winds were all correctly categorized as wave-eroded when subjected to the same statistical procedure as the simulations with isotropic winds. This implies that our ability to identify signatures of wave erosion in coastline roughness does not depend strongly on the assumption of isotropic winds.
