# Supplementary material for: Signatures of wave erosion in Titan’s coasts
Source: Sci Adv. 2024 Jun 19;10(25):eadn4192. doi: 10.1126/sciadv.adn4192 (PMC11186481; doi:10.1126/sciadv.adn4192)
Supplement: Supplementary file 1 — Figs. S1 to S11 Tables S1 to S4 [file sciadv.adn4192_sm.pdf]

Science Advances
AAAS

Supplementary Materials for

Signatures of wave erosion in Titan's coasts

Rose V. Palermo *et al.*

Corresponding author: Rose V. Palermo, rpalermo@usgs.gov

Sci. Adv. **10**, eadn4192 (2024)
DOI: 10.1126/sciadv.adn4192

This PDF file includes:

Figs. S1 to S11
Tables S1 to S4

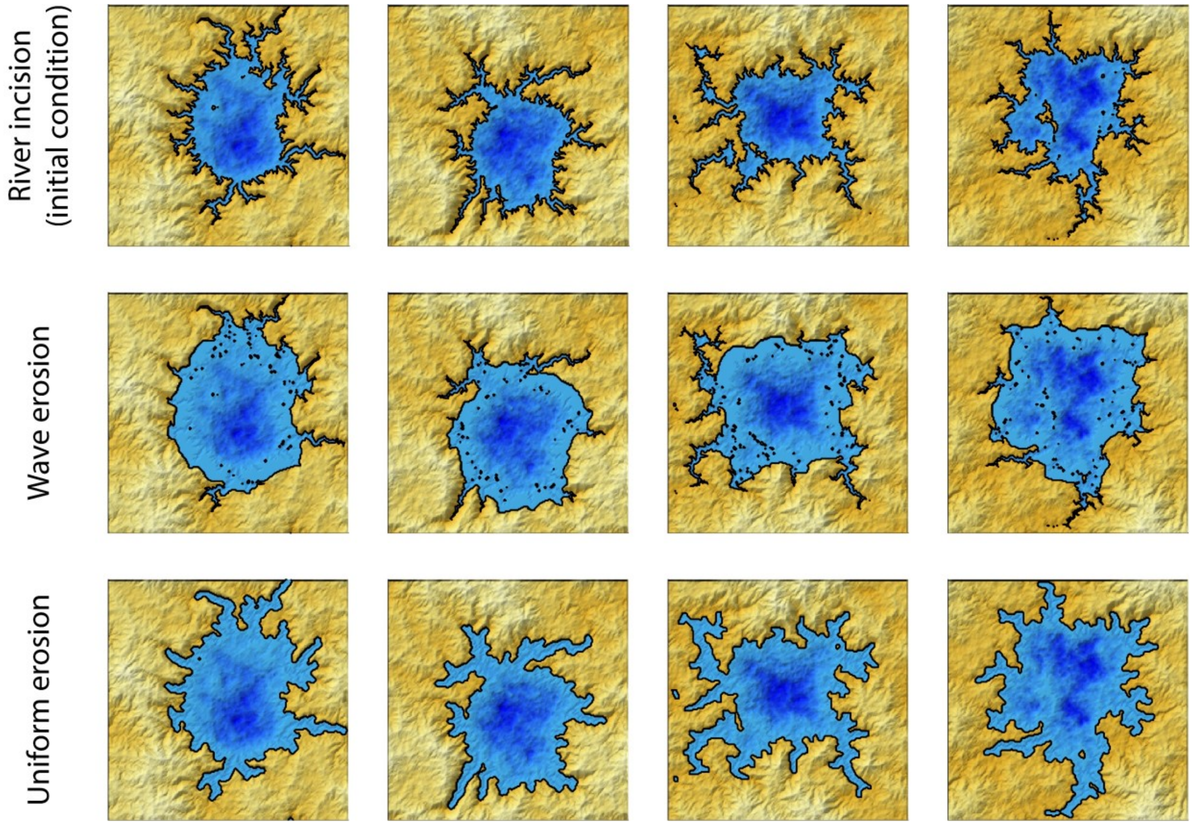

Fig. S1. Model shoreline examples. Examples of synthetic flooded landscapes eroded by river incision with no subsequent coastal erosion (first row), wave erosion (second row), or uniform erosion (third row). Each column the same initial condition landscape.

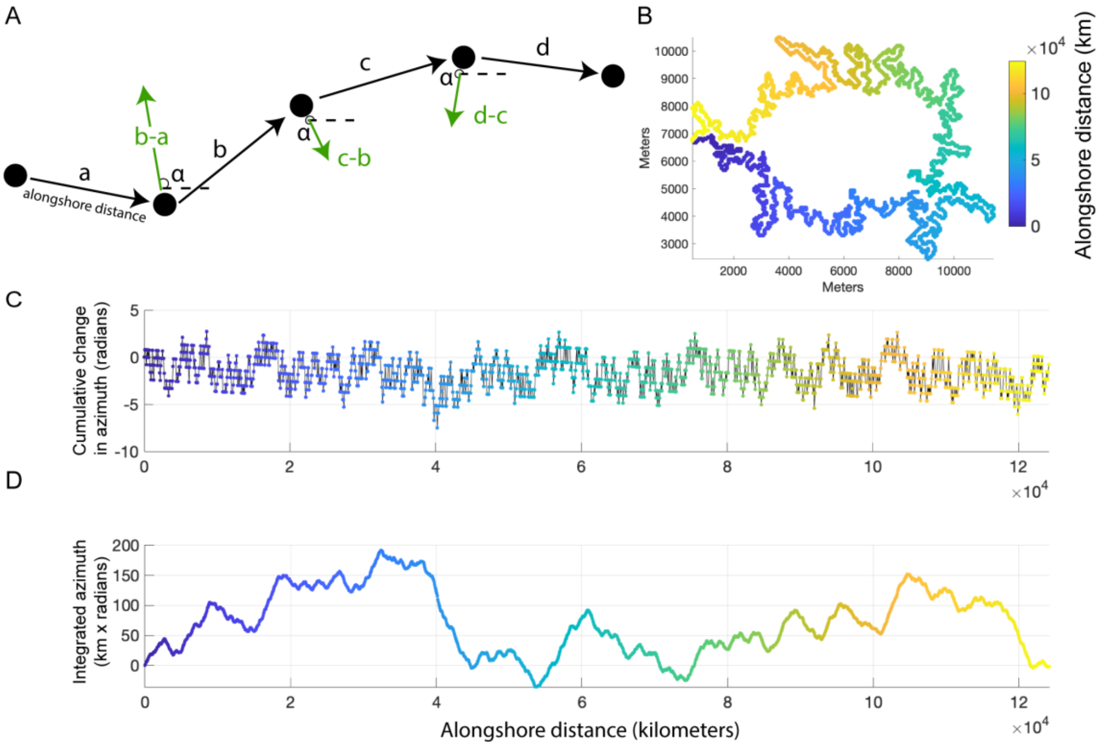

Fig. S2. Illustration of the procedure for "unraveling" a closed shoreline. (A) Schematic showing the measurement of azimuth (anti-clockwise angle, α) and alongshore distance between consecutive vectors connecting shoreline points. (B) Shoreline of an example model initial condition consisting of flooded river valleys (see Fig. 2A), with color indicating alongshore distance. (C) The cumulative change in azimuth between successive shoreline points as a function of alongshore distance, detrended to remove the change in azimuth of 2π radians around the closed shoreline. Before detrending, the azimuth only takes on discrete values in increments of 0.25π radians because of the model grid. Color indicates alongshore distance, as in B. (D) The integral of the detrended cumulative change in azimuth with respect to distance as a function of alongshore distance, representing the "unwrapped" shoreline. Color indicates alongshore position, as in B.

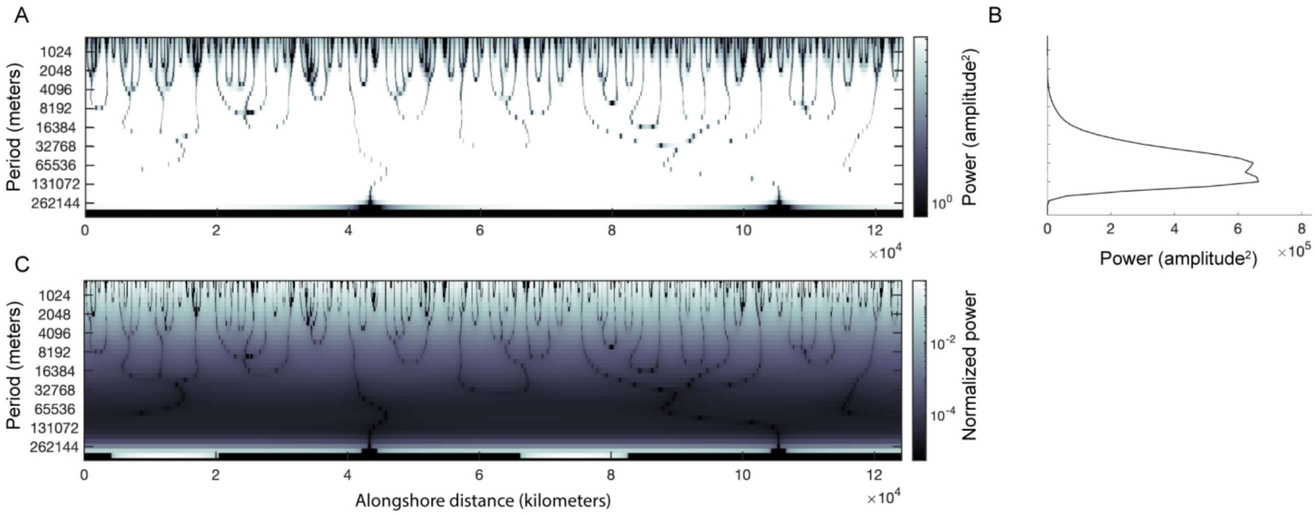

Fig. S3. Wavelet power spectrum. (A) Wavelet power spectrum of the unwrapped shoreline in fig. S1D. (B) Global wavelet power spectrum of the unwrapped shoreline in fig. S1D. (C) Normalized wavelet power spectrum produced by dividing the spectrum for each position in A by the global spectrum in B.

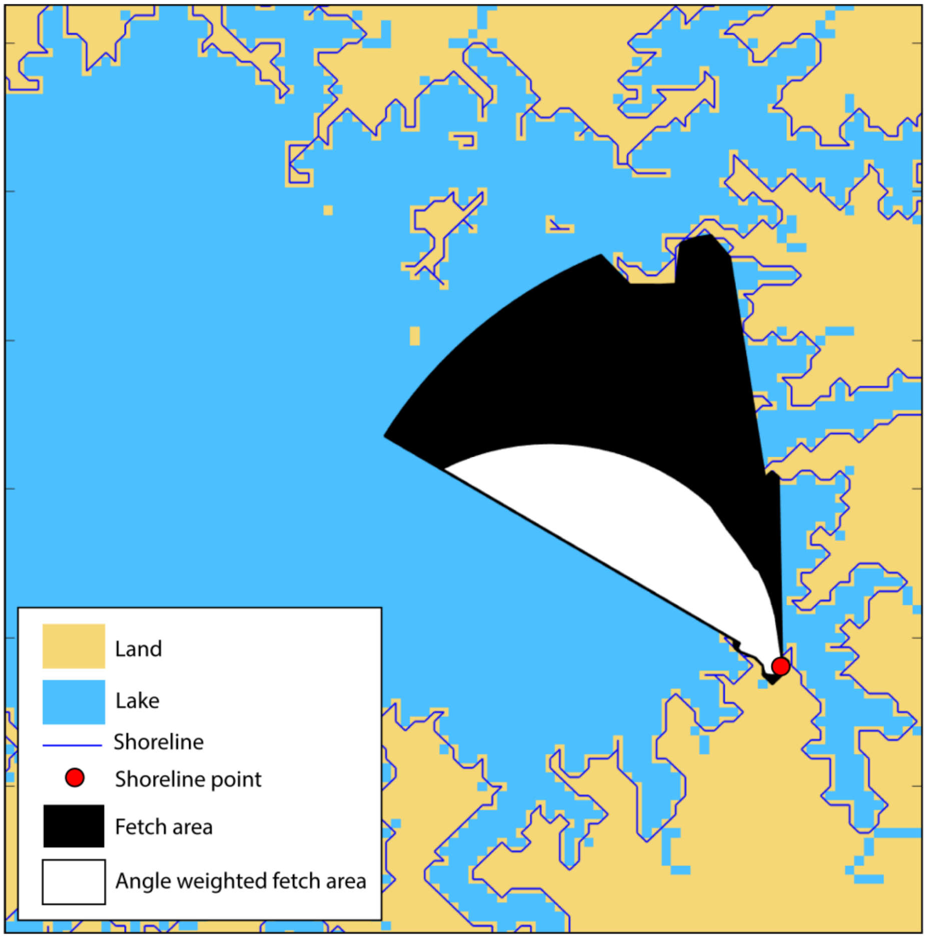

Fig. S4. Illustration of fetch area. Map shows a portion of the 8-connected shoreline of the example in Fig. 2A. For a point along the shoreline (red circle), we show the fetch area (black) and the angle-weighted fetch area (white). Both fetch areas are calculated with a saturation length of 48 grid cells.

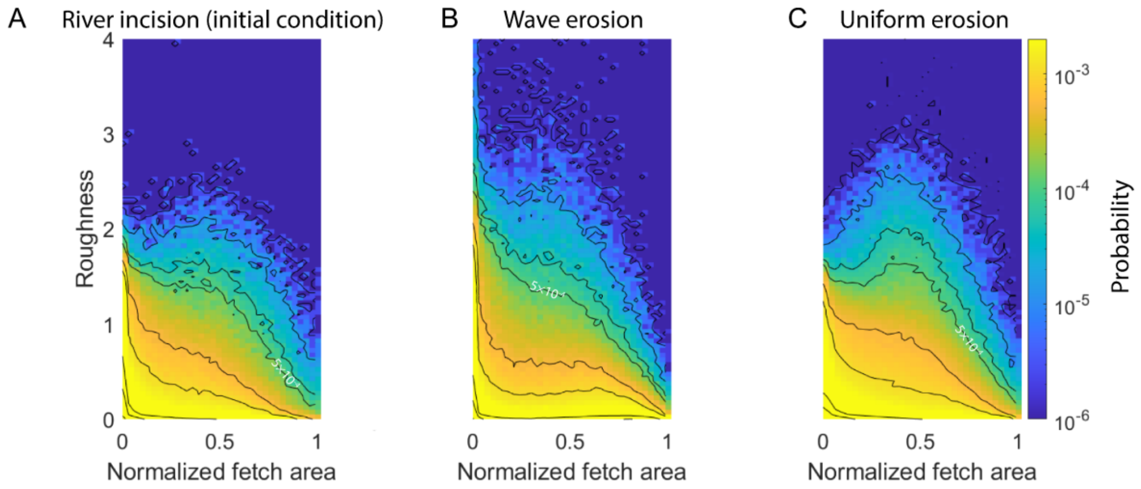

Fig. S5. Joint probability distribution functions (JPDFs) of shoreline roughness and normalized fetch area for all 2305 model simulations of three end-member coastal erosion process scenarios. JPDFs are approximated with two-dimensional histograms. The three scenarios are (A) the model initial condition formed by flooding a landscape that was previously incised by rivers (n=319), (B) simulations of wave erosion after lake area has increased to 120%, 130%, 140%, or 150% of its initial value (n=624), and (C) simulations of uniform erosion after lake area has increased to 120%, 130%, 140%, or 150% of its initial value (n=838). Contours (black lines) for probabilities 1×10^{-6}, 1×10^{-5}, 5×10^{-5}, 1×10^{-4}, 5×10^{-4} (labeled), 1×10^{-3}, 5×10^{-3}, 1×10^{-2}, and 5×10^{-2} are shown to illustrate the shapes of the distributions.

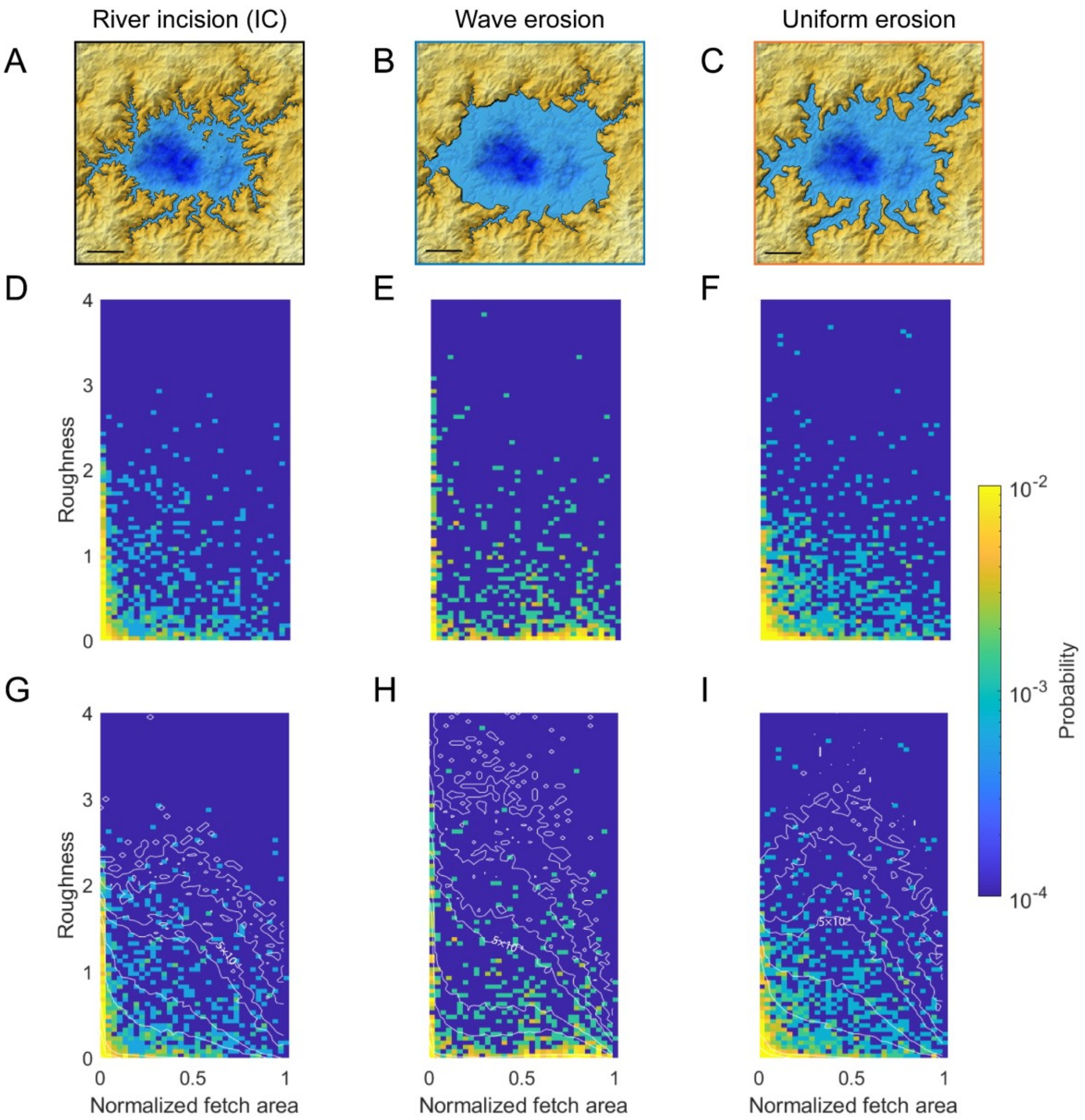

Fig. S6. Joint probability distribution functions (JPDFs) of shoreline roughness and normalized fetch area for example model simulations of three end-member coastal erosion processes. Shoreline maps are the same model simulations shown in Fig. 2: (A) model initial condition formed by flooding a landscape that was previously incised by rivers, (B) simulation of wave erosion after lake area has increased to 150% of its initial value, and (C) simulation of uniform erosion after lake area has increased to 150% of its initial value. JPDFs are approximated with two-dimensional histograms for (D) the shoreline in A, (E) the shoreline in B, and (F) the shoreline in C. The same JPDFs are plotted in (G-I) with the probability contours for the characteristic distributions of the corresponding processes (fig. S4A-C).

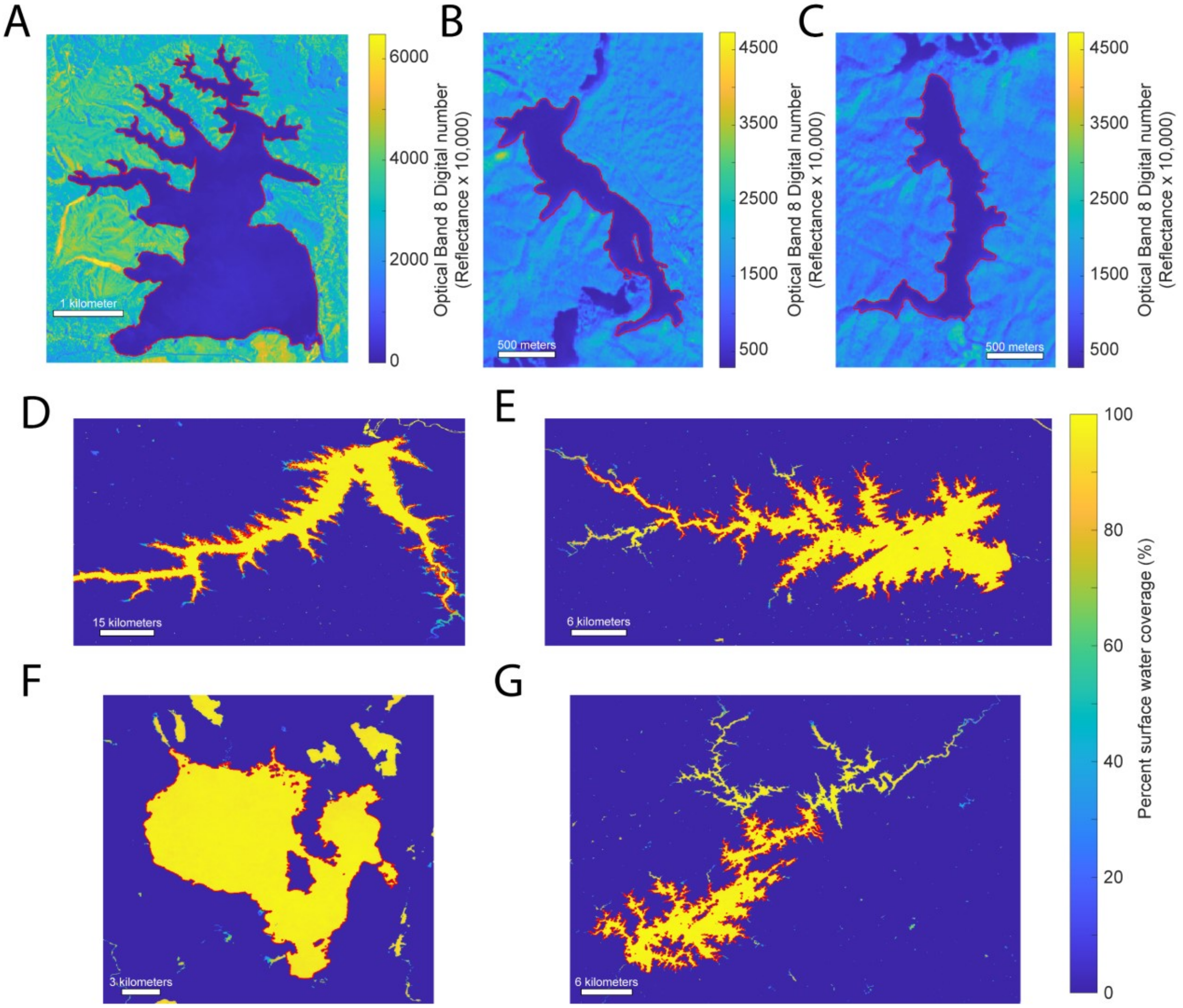

Fig. S7. Mapped lakes on Earth. Shorelines (red) for Earth lakes plotted over (A-C) Band 8 of the associated Sentinel2 image or on (D-G) a map of the percent occurrence of surface water in each pixel (51). Lakes plotted are (A) Lake Rotoehu, New Zealand (NZ); (B) Kozjak Jezero, Croatia (HR); (C) Prošćansko Jezero, HR; (D) Fort Peck Lake, United States of America (USA); (E) Lake Murray, USA; (F) Sebago Lake, USA; and (G) Lake Lanier, USA. See table S4 for image information.

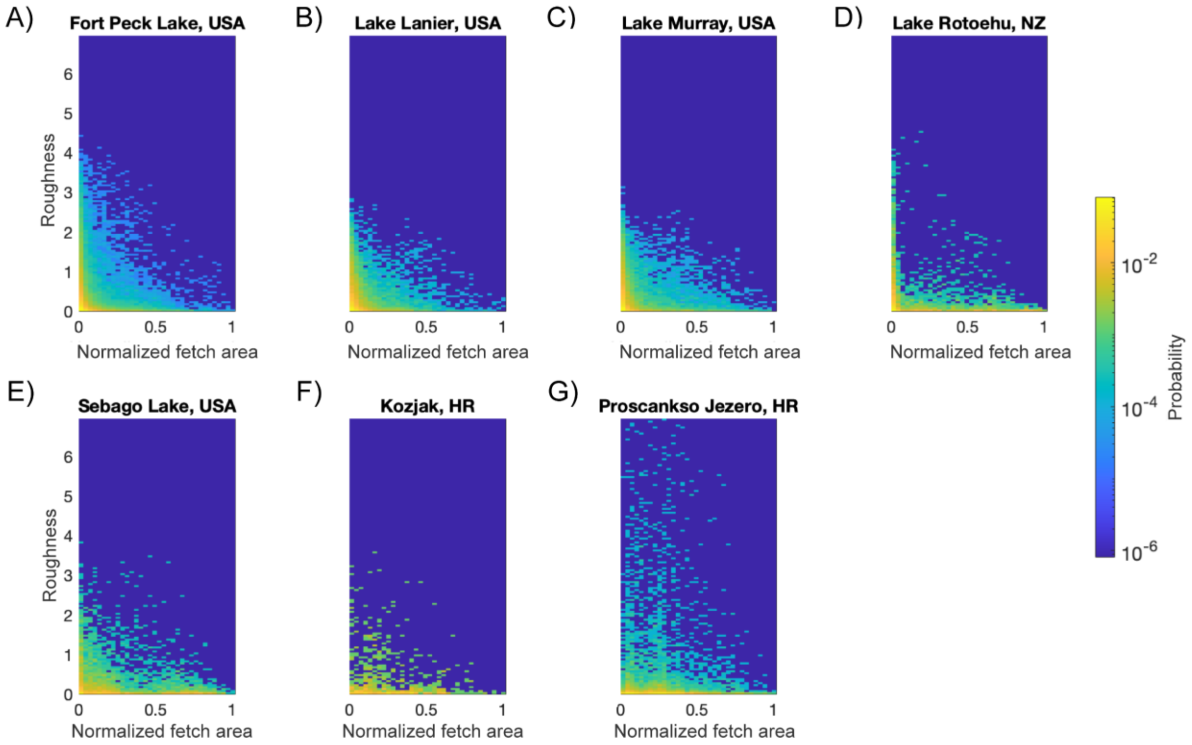

Fig. S8. Joint probability distribution functions of shoreline roughness and normalized fetch area for mapped lakes on Earth. (A) Fort Peck Lake, United States of America (USA); (B) Lake Lanier, USA; (C) Lake Murray, USA; (D) Lake Rotoehu, New Zealand (NZ); (E) Sebago Lake, USA; (F) Kozjak Jezero, Croatia (HR); (G) Proscankso Jezero, HR.

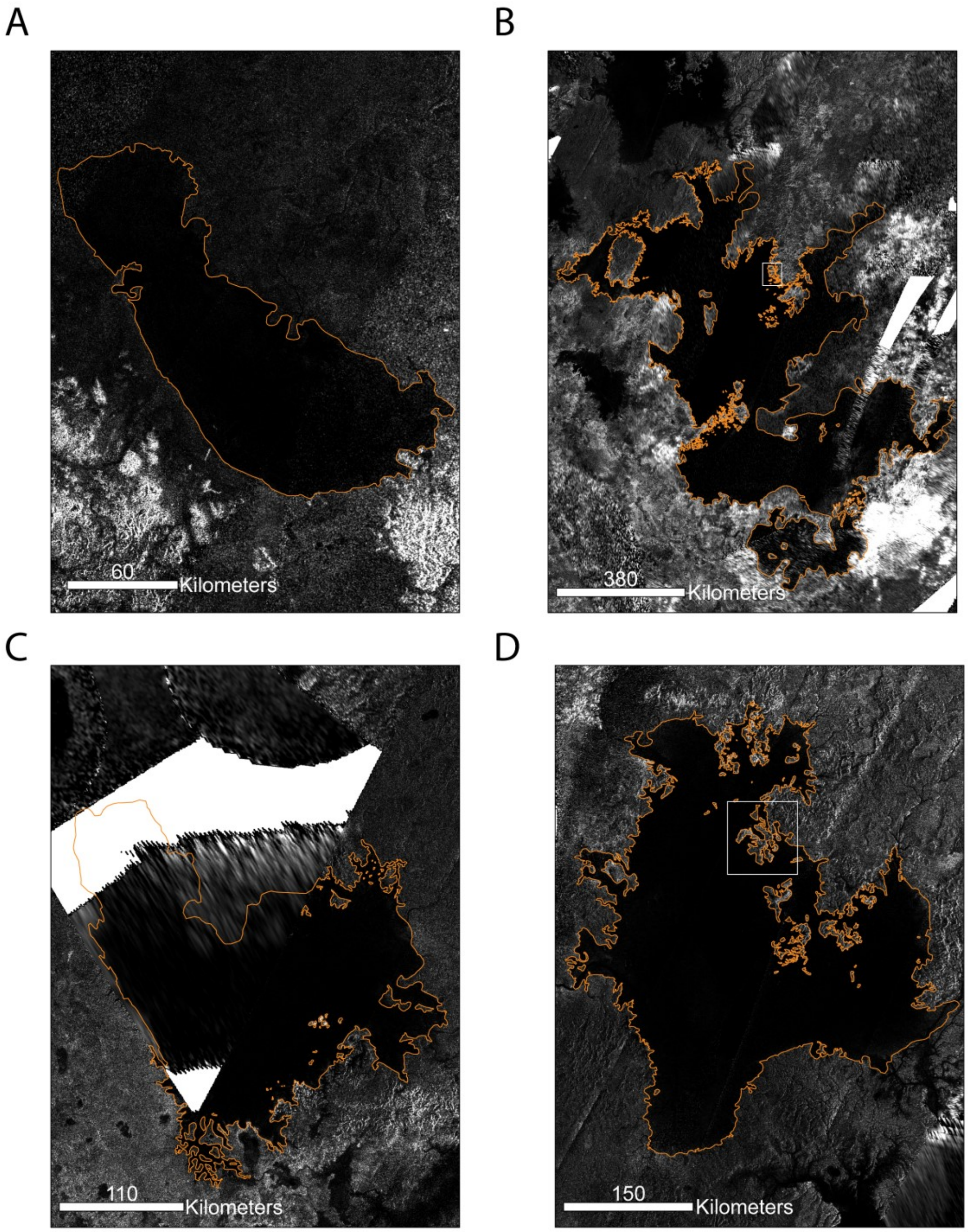

Fig. S9. Mapped lakes on Titan. Shorelines (orange) plotted on Cassini Synthetic Aperture Radar (SAR) imagery for (A) Ontario Lacus, (B) Kraken Mare, (C) Punga Mare, and (D) Ligeia Mare. Inset white boxes in B and D indicate locations shown in Fig. 3. Cassini SAR data is publicly available via the NASA Planetary Data System (https://pds-imaging.jpl.nasa.gov/volumes/radar.html).

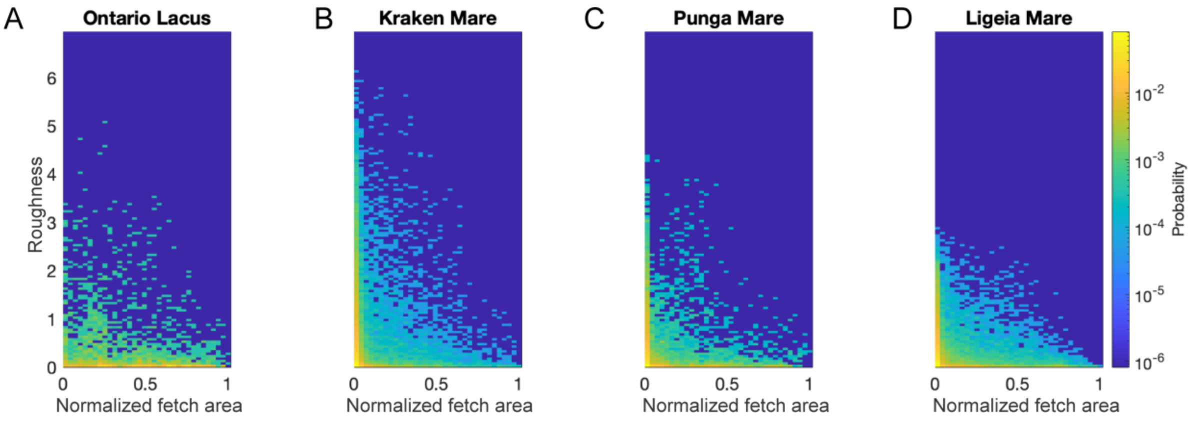

Fig. S10. Joint probability distribution functions of shoreline roughness and normalized fetch area for liquid bodies on Titan. (A) Ontario Lacus, (B) Kraken Mare, (C) Punga Mare, and (D) Ligeia Mare.

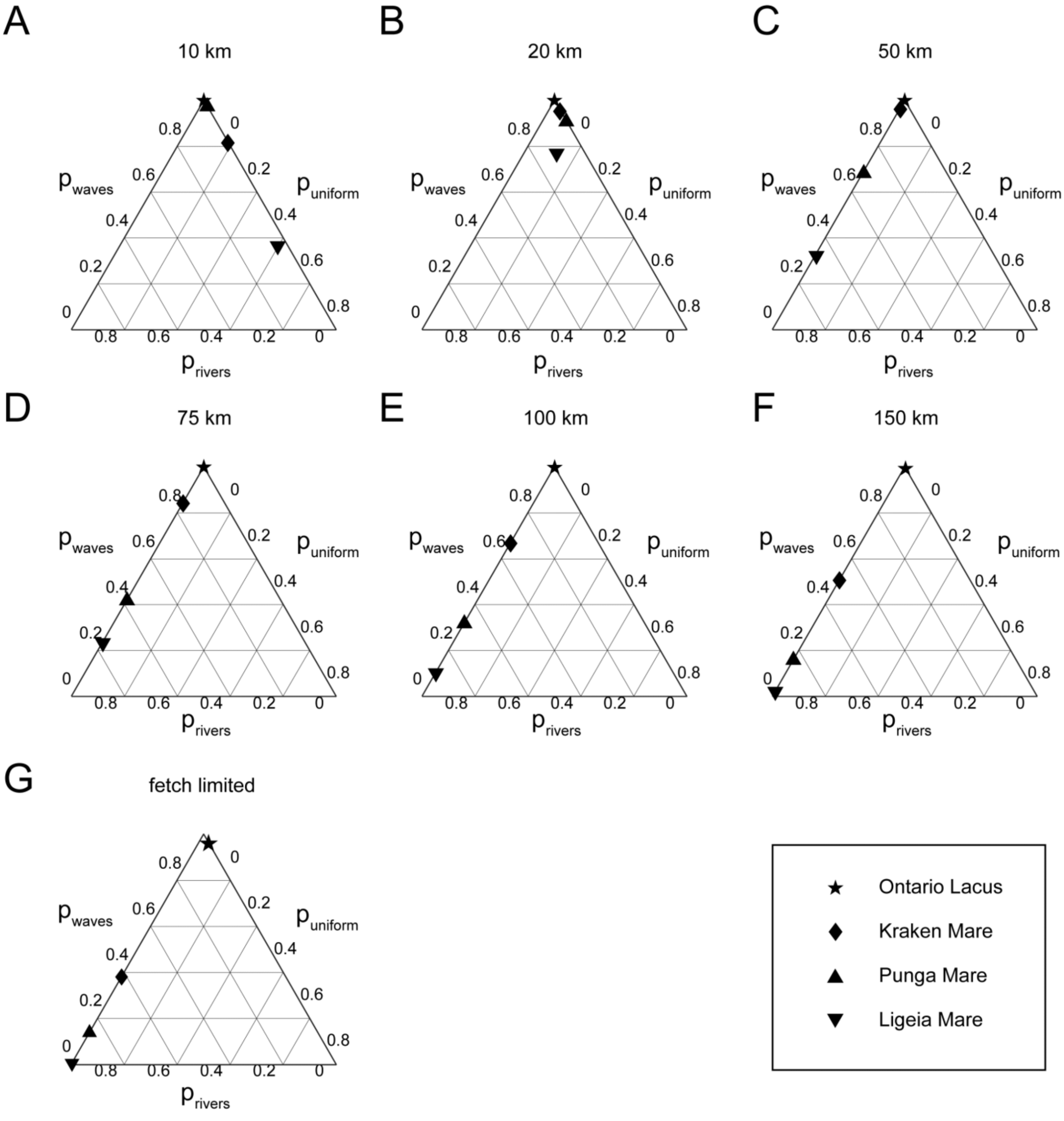

Fig. S11. Ternary diagrams showing probabilities of the three shoreline formation process scenarios for mapped shorelines on Titan for different saturation fetch lengths. Each panel has the same format as Figure 4 but assumes (A-F) waves that saturate at the indicated fetch length, or (G) fetch-limited conditions. (B) is the same as the dark magenta points in Figure 4; (G) is the same as the light magenta points in Figure 4. Figures made using Ternplot (60).

Table S1. Input parameters for numerical model simulations using Numerical model of coastal Erosion by Waves and Transgressive Scarps (NEWTS) (41).

**Parameter**	**Definition (units)**	**Value**
Nx	Number of grid cells in x direction	200
Ny	Number of grid cells in y direction	200
dx	Grid spacing in x direction (m)	62.5
dy	Grid spacing in y direction (m)	62.5
doAdaptiveCoastalTimeStep	Switch for adaptive time step	TRUE
dtmax	maximum time step (yr)	100
size_final	ratio of final to initial basin size that terminates run	1.2, 1.3, 1.4, 1.5
con8	shoreline is 8 connected (1) or 4 connected to adjacent points (0)	TRUE
So	reference strength	1
dxo	reference grid cell size (m)	100
delta	step size for fetch calculation (fraction of a cell)	0.05
nrays	Number of rays in fetch calculation	180
alpha_threshold_strength	Threshold strength parameter	0.1
sealevel_init	Initial sea level (m)	40
deptheroded	Wave base or Depth of uniform erosion (m)	0.5
Kuniform	Uniform erosion rate constant (1/yr)	1.00×10^{-3}
Kwave	Wave erosion rate constant (1/yr)	1.00×10^{-3}

Table S2. P-values for Kruskal-Wallis tests applied to the modeled shorelines for three coastal erosion process scenarios (the river-incised initial condition, wave-eroded shorelines, and uniformly eroded shorelines) and contours from a smooth, random surface for three different shoreline morphology metrics. Of the 439,172 data points, 4,000 were chosen at random for this analysis. Results show that the distributions of normalized fetch area, roughness, and roughness divided by the normalized fetch area for each erosional process scenario and the smooth surface are highly unlikely to have been drawn from the same distribution. This demonstrates that the shorelines for the three erosional process scenarios and the smooth surface are morphologically distinct from one another.

Parameter	Value
Normalized fetch area	5.52×10^{-16}
Roughness	3.67×10^{-269}
Roughness / normalized fetch area	8.04×10^{-169}

Table S3. P-values for Kruskal-Wallis tests applied to wave-eroded shorelines from model simulations that assume an isotropic wave climate and model simulations that assume an anisotropic wave climate. Of the 65,710 data points, 2,000 were chosen at random for this analysis. Results show that it is not possible to reject the null hypothesis that the distributions of normalized fetch area, roughness, and roughness divided by the normalized fetch area for each scenario were drawn from the same distribution. This demonstrates that when a shoreline is eroded to 150% of its initial area using the model parameters investigated in this study, the roughness characteristics of shorelines formed by isotropic and anisotropic wave climates are not statistically distinguishable.

**Parameter**	**Value**
Normalized fetch area	7×10^{-4}
Roughness	0.45
Roughness / normalized fetch area	0.12

Table S4. Data source, band (if applicable), and applied thresholds used for automated mapping of shorelines on Earth in Croatia (HR), New Zealand (NZ), and United States of America (USA). Sentinel-2 data are available at: https://dataspace.copernicus.eu/explore-data/data-collections/sentinel-data/sentinel-2. Google Earth Surface Water data are available at: https://developers.google.com/earth-engine/datasets/catalog/JRC_GSW1_4_GlobalSurfaceWater.

**Lake**	**Data Source**	**Data**	**Band**	**Threshold**
Proscankso Jezero, HR	Sentinel-2	COPERNICUS/S2_SR/20210224T100029_20210224T100025_T33TWK	8	800
Kozjak Jezero, HR	Sentinel-2	COPERNICUS/S2_SR/20210224T100029_20210224T100025_T33TWK	8	800
Lake Lanier, USA	Google Earth Surface Water	(58)	N/A	75%
Lake Murray, USA	Google Earth Surface Water	(58)	N/A	75%
Fort Peck Lake, USA	Google Earth Surface Water	(58)	N/A	75%
Lake Rotoehu, NZ	Sentinel-2	COPERNICUS/S2_SR/20210110T221611_20210110T221605_T60HVC	8	400
Sebago Lake, USA	Google Earth Surface Water	(58)	N/A	20%
